# Cell-free and concentrated pleural effusion reinfusion therapy with aggressive nutritional support improved refractory pleural effusion in a patient with malnutrition after distal gastrectomy for gastric cancer

**DOI:** 10.20407/fmj.2024-030

**Published:** 2025-04-17

**Authors:** Masanobu Usui, Manami Matsumoto, Yoshinori Itani, Norimasa Tsuzuki, Miyo Murai, Akihiro Ito, Akihiko Futamura

**Affiliations:** 1 Department of Surgery and Palliative Medicine, Fujita Health University, School of Medicine, Toyoake, Aichi, Japan; 2 NST, Fujita Health University Nanakuri Memorial Hospital, Tsu, Mie, Japan; 3 Department of Medical Technology, Clinical Examination Division, Fujita Health University Nanakuri Memorial Hospital, Tsu, Mie, Japan

**Keywords:** Refractory pleural effusion, Concentrated pleural fluid filtration reinfusion therapy, Aggressive nutritional support

## Abstract

An 82-year-old man underwent distal gastrectomy for gastric cancer 6 months before admission to our hospital. His postoperative food intake was reduced to less than half of the preoperative amount. Two months postsurgery, he fell and fractured his leg and was bedridden. Furthermore, hypoalbuminemia and bilateral pleural effusions persisted. Despite repeated administration of an albumin preparation and pleural drainage, he showed no improvement for 3 months and was referred to our hospital. On admission, his height was 171 cm, weight 52.8 kg, and body mass index 18.1 kg/m^2^. Moreover, he was clearly undernourished and had difficulty maintaining a standing position. In terms of nutritional enrichment, his diet was adjusted to meet the needs of an older adult patient postgastrectomy. A protein-enriched supplemental diet of approximately 1800 kcal was planned, and he could consume almost the entire amount. His refractory pleural effusion was resolved by performing concentrated pleural effusion reinfusion therapy, and he continued to receive aggressive nutritional support and rehabilitation. The patient’s general condition and activities of daily living improved markedly. Subsequently, he was discharged and could walk independently on day 64 of hospitalization. Summary: We experienced a successful case of refractory pleural effusion due to malnutrition treated with aggressive nutritional support combined with concentrated pleural fluid filtration reinfusion therapy.

## Introduction

Cell-free and concentrated ascites reinfusion therapy (CART) is a treatment method in which ascites is filtered and concentrated before reinfusion.^1^ Owing to its minimal invasiveness and expected reduction in nutrient loss due to water withdrawal, CART is considered an effective treatment option for fluid retention caused by malnutrition.^2^ However, there are only a few detailed reports on cell-free and concentrated pleural effusion reinfusion therapy (CPRT) for refractory pleural effusions.

Blood tests and bioelectrical impedance analysis (BIA) are used to objectively evaluate nutritional status. Furthermore, attention has been drawn to the fact that measurement of these nutritional indices can be used not only to predict prognosis at the terminal stage of cancer but also evaluate nutritional improvement.^3^

Herein, we reported and objectively evaluated a case of refractory pleural effusion associated with malnutrition that was successfully treated with CPRT and aggressive nutritional support.

## Case

An 82-year-old male.

Chief complaint: refractory pleural effusion, malnutrition, disuse syndrome

Medical history: Unremarkable

History of present illness: Six months before admission to our hospital, the patient was diagnosed with early gastric cancer (greater curvature of the gastric body IIc+pyloric lesion, cT1N0M0; cStageIA) and underwent distal gastrectomy with D1 lymph node dissection. He underwent Roux-en-Y reconstruction for early stage gastric cancer because there were two lesions in the stomach and it was difficult to perform Billroth I reconstruction on the proximal side due to the distance. The surgery time was 4 hours and 35 minutes, and the volume of blood loss was 300 mL. Pathological findings were well-differentiated tubular adenocarcinoma, pT1a(M), ly0, v0, PM(–), DM(–) on the oral side. The pyloric lesion was identified as poorly differentiated adenocarcinoma, pTib2(SM2:1.2mm), INFβ, ly0, v0, PM(–), DM(–). Lymph nodes were N0.3: (–) [0/1], N0.7 (–) [0/7]. There was no lymph node metastasis, and the final diagnosis was pT1N0M0; stage IA. Owing to his advanced age and the fact that the gastric cancer was at an early stage, postoperative adjuvant chemotherapy was not administered immediately postsurgery. Despite the limited food intake, the patient strongly desired to be discharged home and was followed up at the outpatient clinic of the previous hospital. Immediately postsurgery, the amount of food intake decreased, and portioned meals were provided. The patient was considered malnourished, as his 1-month postoperative food intake reduced to less than half of the preoperative amount. At 2 months postsurgery, the patient was hospitalized and confined to bed rest due to a sacral fracture caused by a fall. He developed sepsis from aspiration pneumonia, requiring intensive care and long-term ventilator management. During this period, oral intake was not possible because the patient was intubated, and enteral nutrition could not be supplemented adequately due to his impaired gastrointestinal motility. Therefore, he had to rely on intravenous central nutrition, leading to malnutrition and disuse syndrome. After being weaned from respiratory ventilation, hypoalbuminemia and bilateral pleural effusions persisted. Following extubation, the patient could eat and was provided with 800 kcal of soft vegetables. However, due to irregularity in food intake and a small residual stomach, there was limited food intake, which was divided into small portions. The patient weighed 65 kg preoperatively. However, at the time of discharge postoperatively, he weighed 52 kg. He could not eat well at home. Upon the second admission to the previous hospital, he weighed 54 kg (showing a weight loss of >10 kg) and presented with pleural effusion. The actual food intake was 20%–30% of a 1600 kcal-diet consisting of soft vegetables, which were provided in separate portions. Central venous nutrition was not attempted, and approximately 500 kcal/day was administered peripherally. The patient was administered a hypertonic albumin preparation, and pleural drainage was performed every 2 days. However, even after 3 months of continuous treatment, weight gain was not observed, hypoalbuminemia was 2.0 g/dL, and the pleural effusion did not improve. Consequently, the patient was referred to our hospital with a diagnosis of intractable pleural effusion due to malnutrition.

Condition at admission: The patient had a height of 171.0 cm, weight of 52.8 kg, and body mass index of 18.1 kg/m^2^. Mild anemia was observed in the conjunctiva, but there was no jaundice. The patient was extremely thin and could not stand up from a wheelchair independently. Dyspnea was noted, and drainage tubes were placed on both sides of the thoracic cavity. The pleural fluid was pale yellow and clear, and the cytology results were negative.

Hematological findings on admission: White blood cell count, 3,150/μL; total lymphocyte count, 1,880/mm^3^; hemoglobin level, 9.3 g/dL; total protein content, 5.3 g/dL; and albumin level, 2.2 g/dL. Low white blood cell and lymphocyte counts, anemia, and hypoalbuminemia were noted. The levels of C-reactive protein, transthyretin (TTR), magnesium, phosphorus, copper, zinc, and lactic acid were 0.3 mg/dL, 20.1 mg/dL, 1.9 mg/dL, 3.4 mg/dL, 80 μg/dL, 68 μg/dL, and 11 mg/dL, respectively.

**Figure 1:**
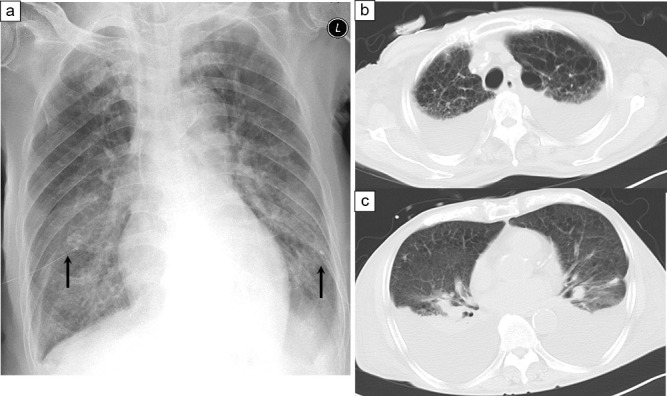
Imaging study on admission. a: Chest X-ray (supine position); b, c: Chest CT. a: Pleural effusion and thoracic drainage tubes were placed on both thoracic cavities (black allow). b, c: Moderate amounts of pleural effusion were observed on both sides.

Imaging findings at admission:

Chest X-ray (Figure 1a): Uniformly decreased permeability of the lung fields was observed in the bilateral thoracic cavities, with bilateral pleural effusions and drainage tubes placed on both sides of the thoracic cavity by the previous physician.

Chest computed tomography (CT) (Figure 1b,c): A moderate amount of pleural effusion was noted bilaterally (after drainage on the previous day).

Post-hospitalization course: During subjective global assessment, the patient was evaluated as severely malnourished, and the predicted energy requirement was calculated as 1573 kcal (basal metabolic rate [1093 kcal]×activity coefficient [1.2]×stress coefficient [1.2]). Accordingly, the basic plan was set, and the patient consumed 1600 kcal of energy, 65 g of protein (including 2 g of branched-chain amino acid [BCAA]), 45 g of fat, and 230 g of carbohydrates (protein:fat:carbohydrate [PFC] ratio=16:25:58) per day. Owing to the patient’s poor appetite after gastrectomy, meals were set to ensure that half of the diet comprised soft vegetables (800 kcal, 35 g protein). The diet was adjusted to meet the patient’s preferences, and the patient could consume the entire amount from the beginning of his hospitalization. A peripherally inserted central catheter was placed, through which the diet deficiency was overcome by supplementation with central intravenous nutrition (high-calorie infusion+fat emulsion: 760 kcal, 20 g of amino acids), and half a packet of oral nutritional supplements (ONS) (150 kcal, 4.4 g of protein) was added to enrich with amino acids and protein. The daily dose was 1710 kcal energy (950 kcal oral+760 kcal IV), 59.4 g of protein (5.3 g of BCAA, 8.9%), 40 g of fat, and 273 g of carbohydrates (PFC ratio=14:21:65). CPRT was performed for bilateral pleural effusions because chest tubes were already in place for bilateral pleural effusions with dyspnea and could be safely drained.

**Figure 2:**
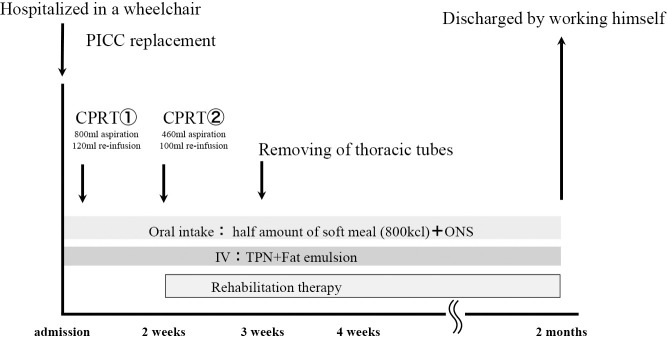
Progress after admission CPRT: Cell-free and concentrated pleural effusion reinfusion therapy

**Figure 3:**
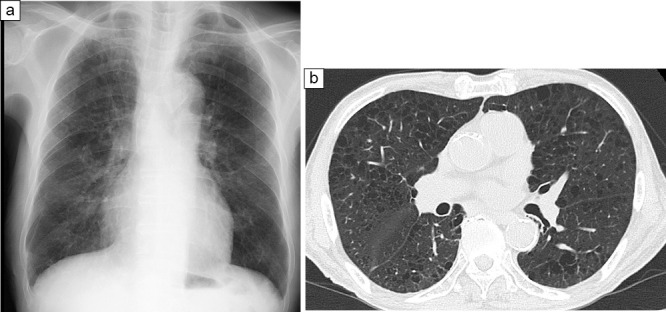
Imaging study 2 months after admission: a: Plain chest X-ray (standing position). b: Chest CT scan. a, b: No pleural effusion was observed on either side.

During the first CPRT, 800 mL of bilateral pleural fluid was collected, and 120 mL was returned. During the second CPRT performed 2 weeks later, 460 mL of bilateral pleural fluid was collected, and 100 mL was returned. The drained effusions were returned after cytological examination to confirm the absence of leukocytosis as well as bacterial, serum, and biochemical problems. Six weeks later, the chest tubes were removed because the effusions had disappeared. The patient’s general fatigue improved after the second CPRT, and rehabilitation therapy was started 2 weeks after admission (Figure 2). Evaluation at the start of rehabilitation therapy revealed that the patient was unsteady and could not walk. In addition, edema was observed in both lower limbs. The result of manual muscle test (MMT) was level 4; moreover, for activities of daily living (ADL), the functional independence measure (FIM) revealed a score of 4 for self-care and 3 for transfer ability. Accordingly, wheelchair transfers were initiated. Weekly rounds were conducted by the nutritional support team, and a nutritional treatment plan was evaluated. During this period, it was difficult to increase his food intake due to dumping symptoms, and concurrent intravenous nutrition was used. However, as there was no weight loss and no elevation of liver function or neutral fat as per blood tests, it was judged that there was no excess or deficiency of the nutritional diet administered, and the nutritional plan developed at the time of hospitalization was continued. Among the nutritional indicators, serum TTR increased from 20.7 to 27.2 mg/dL within 2 weeks after admission, and serum albumin level gradually increased from 2.2 g/dL at admission to 3.0 g/dL 2 months later. Serum lactate levels also decreased from 11.2 to 7.3 mg/dL. Two months after admission, plain chest radiography and CT revealed no bilateral pleural effusions (Figure 3). Furthermore, body composition wasmeasured immediately after admission and 2 months laterusing a body component analyzer (InBody^TM^). The phase angle was 2.0° at the time of admission when edema was present, but it increased to 2.9° 2 months later. Moreover, the extracellular water (ECW)/ total body water (TBW) ratio, an indicator of edema, showed a slight improvement from a high value of 0.436 (>0.43) to a slightly low value of 0.423 (>0.40 to <0.43) (Table 1). Subsequently, no reaccumulation of pleural effusion was observed, and walking practice was performed during rehabilitation therapy. The final MMT result was level 5 (normal), indicating the ability to move through the entire range of motion even with strong resistance. Regarding ADL, the FIM score was 6 points for self-care, 7 for transfer, and 7 for locomotion, indicating that he could walk independently without a cane. Finally, the patient was discharged from the hospital.

After discharge from the hospital, the patient did not wish to continue the supplemental intravenous nutrition being provided. As part of the nutritional management plan after discharge from the hospital, we investigated whether it would be possible to treat him with oral intake alone. The predicted energy requirement after discharge was calculated as 1429 kcal (basal metabolic rate [1099] kcal×activity coefficient [1.3]×stress coefficient [1.0]). Nutritional guidance was provided at the time of discharge with a plan designed to ensure that the patient would consume 1400 kcal of energy, 60 g of protein, 40 g of fat, and 200 g of carbohydrate (PFC ratio=17:26:57). During hospitalization, three meals and supplemental meals of ONS at 10:00 and 15:00 were proposed to the patient’s family. The patient was discharged on day 64 of hospitalization with unassisted walking. He was allowed to eat small, frequent meals. After being discharged from the hospital, he had to visit his previous doctor’s office immediately based on the assumption that he would be able to receive care when necessary.

## Discussion

Early implementation of enteral or intravenous nutrition, a treatment option for hypoalbuminemia, is effective because the administration of amino acids facilitates protein synthesis.^4^ Our patient showed an improvement upon supplementation with ONS containing BCAA in addition to oral nutritional diet from the early stage of illness.

CART was developed by Yamazaki et al. in Japan in 1973.^5^ It is currently performed for liver cirrhosis and cancer ascites.^2^ CART is mostly used for treating patients with terminal cancer, and few medical institutions perform CART for refractory ascites in malnourished patients.^6^ If the pleural effusion is due to malnutrition, there is a high possibility of improvement with early and appropriate nutritional management, and CPRT is rarely indicated immediately. However, in older patients who require constant drainage, such as in our patient, CPRT is notable as an aggressive treatment for pleural effusion. This is because it is necessary to eliminate the need for drainage at an early stage and allow recovery to a point where daily life can be maintained. A PubMed search for the term “cell-free and concentrated pleural effusion reinfusion therapy,” regardless of the year of publication, yielded two cases, one of which was associated with malnutrition. A search of the Japan Medical Abstracts Society database for the terms “nutritional disorders” and “refractory pleural effusions,” regardless of the year of publication, revealed no articles, and a search for the terms “CPRT,” “concentrated thoracoabdominal effusion reinfusion therapy,” and “concentrated thoracoabdominal fluid reinfusion therapy” revealed three articles on pleural effusions. In addition to these reports, a search of the reference literature revealed a total of six reports of CPRT, as shown in Table 2.^7–12^ However, there were only three cases in which malnutrition was a potential causative factor.

The Japanese Society for Palliative Medicine recommends that the amount of drainage should be approximately 1000 mL.^13^ In contrast, CPRT can increase intravascular osmotic pressure, allowing large amounts of drainage at once. As no pleural effusion remains, symptoms such as dyspnea and general malaise are relieved, resulting in an improvement in ADL. Although some physicians have recommended not to drain >1500 mL of fluid within 24 h, there is little evidence regarding a direct correlation between the amount of fluid drained and the risk of re-expansion pulmonary edema, according to the abovementioned report. Thus, we attempt to drain as much as possible at our department.^13^

Except for a case of intrapleural dissemination of lung cancer^10^ and a case of a chylothorax associated with chest tube failure,^9,12^ case reports in which malnutrition was a causative factor showed that up to two CPRT cycles were used until effective,^7,8,11^ and it was suggested that improvement can be expected after approximately two cycles of CPRT for pleural effusions due to malnutrition, as observed in our case report. Furthermore, in our case, serum TTR level increased from 20.7 to 27.2 mg/dL within 2 weeks, and an early improvement in nutritional status was believed to be a factor associated with completing CPRT within two sessions. Regarding complications, previous reports have described pneumothorax occurring in patients who underwent frequent paracentesis, but no complications were reported in patients who underwent pleurodesis up to 2 times as in the present case, suggesting that CPRT can be performed safely. Although some institutions administer steroids as a prophylactic measure during perfusion of ascites, we do not administer steroids prophylactically in our department due to fluctuations in blood glucose levels and immunological considerations. Moreover, most of our patients are those with terminal cancer or immunocompromised patients with a poor nutritional status. In our department, there was only one case of mild pneumothorax caused by frequent paracentesis, but the patient had no problem with conservative treatment. There have been no other cases of pneumothorax, and treatment with antipyretics has been administered without any issues, with only a slight fever reported. CPRT for refractory pleural effusion due to malnutrition is an intervention used in combination with aggressive nutritional support, and the effects of respiratory symptoms and nutritional improvement due to decompression drainage by CPRT may have been additive.

Recently, in stages such as precachexia, where metabolic abnormalities and malnutrition have not progressed, resistance exercise to maintain or increase muscle mass has been recommended.^14,15^ In the present case, the patient could walk unassisted after rehabilitation and was discharged from the hospital. After rehabilitation, his MMT improved from 4 to 5, his FIM score from 4 to 6 for self-care and from 3 to 7 for transfer assistance, and his mobility improved from being unable to stand to 7 for independent walking. Although SMI decreased slightly from 7.09 to 6.85 in terms of muscle mass, an improvement in muscle strength was observed. The phase angle measured by BIA indicates the state of cell membranes and is used as a biomarker of malnutrition.^16^ It is defined as the phase difference between the resistance generated when electric current flows along the body water (resistance) and the resistance generated when it passes through the cell membrane (reactance). In other words, the phase angle reflects the health of the cell membrane and the degree of structural stability of the cell, with a phase angle of 0° indicating cell destruction. Therefore, the lower the phase angle, the lower the health and function of the cell.^17^ In the present case, the phase angle improved from 2.0° to 2.9°; however, it is not possible to judge whether it is effective or not. ECW/TBW also showed a slight improvement from high to slightly high. It is useful to judge whether patients with high extracellular water content and low phase angle on electrical impedance at the time of admission, as observed in the present case, show improvement over time. Aggressive nutritional support, CPRT, and rehabilitation therapy were considered to be effective methods in this case report, which led to the patient’s discharge from the hospital in an ambulatory state.

## Figures and Tables

**Table 1:** Changes in nutritional assessment have obtained from the body composition analyzer (InBody^TM^). Phase angle and ECW/TBW have been improved

	Phase angle	ASM (kg)	SMI (kg/m^2^)	ECW/TBW
admission (edema+)	2.0°	20.73	7.09	0.436
2 months later	2.9°	20.02	6.85	0.423

ASM: appendicular skeletal Muscle. SMI : skeletal muscle index. ECW/TBW : extracellular water/total body water

**Table 2:** List of case reports of cell-free and concentrated pleural effusion reinfusion therapy

No	Author	Year	Primary disease	Origin	Frequency of CPRT
1	Furukawa, et al.^7^	1990	Hepatocellur carcinoma	Hepatic pleural effusion	2
2	Kurioka, et al.^8^	2003	OHSS*	OHSS*	1
3	Sawada, et al.^9^	2009	Thoracic aneurysmm	chylothorax	7
4	Takeuchi, et al.^10^	2016	lung cancer	pleural dissemination	18
5	Xiaolin, et al.^11^	2021	Esophageal cancer	malnutrition	1
6	Kuwahara, et al.^12^	2024	SLE*	chylothorax	Frequently

* OHSS:ovarian hyperstimulation syndrome * SLE: Systemic lupus erythematosus

## Summary

CPRT and aggressive nutritional support may be effective in resolving effusions and improving ADL and nutritional status in patients with refractory pleural effusions associated with low nutritional status.

## Supplementary Material

PDF-Japanese
