# Supplementary material for: Cell-free and concentrated pleural effusion reinfusion therapy with aggressive nutritional support improved refractory pleural effusion in a patient with malnutrition after distal gastrectomy for gastric cancer
Source: Fujita Med J. 2025 Apr 17;11(3):146–50. doi: 10.20407/fmj.2024-030 (PMC12327212; doi:10.20407/fmj.2024-030)
Supplement: Supplementary file 1 — PDF-Japanese [file fmj-11-146-s001.pdf]

Case report

タイトル：胸水濾過濃縮再静注法と強化型栄養療法により胸水が消失した胃癌術後低栄養による難治性胸水の１例.

ランニングタイトル：難治性胸水に対する濾過濃縮再静注法と強化型栄養療法を行った1例

[1]藤田医科大学医学部　外科・緩和医療学講座

[2]藤田医科大学　七栗記念病院　NST

[3]藤田医科大学　七栗記念病院　臨床検査・輸血課

臼井正信[1]　松本真奈美[2]　井谷功典[3]　都築則正[1]　村井美代[1]　伊藤彰博[1]　二村昭彦[1]

Masanobu Usui, MD, PhD [1], Manami Matsumoto[2], Yoshinori Itani[3], Norimasa Tsuzuki, MD, PhD [1], Miyo Murai, MD, PhD [1], Akihiro Ito, MD, PhD [1], Akihiko Futamura, PhD [1]

[1]Department of Surgery and Palliative Medicine, Fujita Health University, School of Medicine, Toyoake, Aichi, Japan

[2]NST, Fujita Health University Nanakuri Memorial Hospital, Tsu, Mie, Japan

[3]Department of Medical Technology, Clinical Examination Division, Fujita Health University Nanakuri Memorial Hospital, Tsu, Mie, Japan

Case Report

Corresponding author: Usui Masanobu, MD, PhD

Department of Surgery and Palliative Medicine, Fujita Health University,

School of Medicine, 1-98 Dengakugakubo, Kutsukakecho, Toyoake, Aichi

470-1192, Japan

Tel: 0562-93-2111

E-mail: masanobu.usui@fujita-hu.ac.jp

Abstract

82歳男性．当院入院6か月前に胃癌にて幽門側胃切除術を受け，術後摂食量は術前の半分以下に減少した．術後2か月には転倒骨折しベッド上安静となり，その後，低アルブミン血症と両側胸水が遷延した．アルブミン製剤投与と胸水抜水が繰り返されたが，3か月間改善なく当院紹介となった．入院時現症：身長171cm．体重52.8 kg．BMI 18.1 kg/m^2．明らかな低栄養を呈し，立位保持困難であった．栄養強化面からは，高齢者の胃切除術後であることを意識して食事調整を加え，たんぱく質源を強化した補助栄養で構成された約1800 kcalが計画され，ほぼ全量が実投与可能であった．難治性胸水は胸水濾過濃縮再静注法の実施で消失し，以後強化型栄養療法とリハビリテーション治療を継続．全身状態とADLが顕著に改善し，入院第64日目に独歩退院した．【まとめ】低栄養による難治性胸水に対し，胸水濾過濃縮再静注法を併用した強化型栄養療法の有効例を経験した．

Key word：難治性胸水，胸水濾過濃縮再静注法，強化型栄養療法

【はじめに】

腹水濾過濃縮再静注法（cell-free and concentrated ascites reinfusion therapy: CART）は，腹水を濾過濃縮後に再静注する治療法であり[1]，侵襲度の低さと抜水による栄養成分の損失の低減が見込めることから低栄養を原因とする体液貯留の治療法として有効な選択肢と考えられる[2]．しかし難治性胸水に対する胸水濾過濃縮再静注法（cell-free and concentrated pleural effusion reinfusion therapy :CPRT）については詳細な報告は少ない．

栄養状態を客観的に評価する方法として，血液検査や生体インピーダンス法（bioelectrical impedance analysis:BIA）などの測定が実施されており，これら栄養指標の測定ががん終末期の予後予測だけでなく，栄養改善における評価にも使用できることが注目されている[3]．

今回，低栄養に伴う難治性胸水に対しCPRTと強化型栄養療法が著効した１例を経験したので，これら客観的評価とともに報告する．

【症例】82歳男性.

主訴：難治性胸水，低栄養，廃用症候群

既往歴：特記事項なし

現病歴：当院入院6か月前，早期胃癌(胃体部大弯IIc＋幽門病変、cT1N0M0; cStageIA)の診断で幽門側胃切除術，D1リンパ節郭清が行われた．術式は早期胃がんに対して胃内２箇所に病変があり，口側の方は体部でBillroth I法再建は距離的に困難と判断してRoux-enY再建を行った．手術時間は4時間35分，出血量300mlであった．病理所見は，口側がwell differentiated tubular adenocarcinoma, pT1a(M), ly0, v0, PM(-), DM(-). 幽門側病変は, poorly differentiated adenocarcinoma, pTib2(SM2:1.2mm), INF β , ly0, v0, PM(-), DM(-). リンパ節はN0.3: (-)［0/1］，N0.7 (-)［0/7］とリンパ節転移はなく最終的にpT1N0M0; Stage IAであった．高齢の早期胃癌であり術後補助化学療

法は行わず，食事量は少ないが自宅への退院希望が強く前医外来にて経過観察となった．術直後から摂食量少なく分食で提供していた．術後1か月頃から術前の半分以下で推移し，低栄養状態にあったと考えられた．自宅では分食などの食事提供がなされておらず，低栄養状態が続き，術後2か月に転倒による仙骨骨折のため入院となりベッド上安静となった．誤嚥性肺炎から敗血症を発症したため集中治療を要し，長期間の人工呼吸器管理が実施された．この間，挿管中のため経口摂取はできず，経腸栄養も消化管運動機能が低下していたため十分な補充ができず，中心静脈栄養点滴に頼らざるをえず，低栄養状態，廃用症候群に至り，呼吸器離脱後には低アルブミン血症と両側胸水が遷延した．抜管後は，食事摂取可能となり800kcalの軟菜が提供されていたが，食事摂取にムラがあることと残胃が小さいため1回の摂取量が少なく分食で提供されていた．体重は術前65kgであったが術後52kgで退院となり，自宅での食事摂取も進まず2回目の前医入院時は胸水を含めて54kgと10kg以上減った状態であった．食事摂取は1600kcalの軟菜が分食で提供されていたが2〜3割の摂取となっていた．中心静脈栄養は試されておらず，末梢から１日500kcal程度の投与がされていた．また，高張アルブミン製剤の投与と2日に1度の胸水抜水が行われたが，3か月間の継続でも体重増加は認めず，低アルブミン血症は2.0g/dLで胸水貯留は改善せず，低栄養に伴う難治性胸水の診断で当院へ紹介入院となった．

入院時現症：身長 171.0 cm，体重 52.8 kg，body mass index（BMI）18.1 kg/m^2．結膜に軽度貧血を認めたが黄疸は無く，極度に痩せており自力で車いすから立ち上がれず，呼吸困難があり胸腔両側にドレナージチューブが留置されていた．胸水は淡黄色，清であり細胞診は陰性であった．

入院時血液学的検査所見: 白血球数 3,150/μL, 総リンパ球数 1,880/mm^3, ヘモグロビン 9.3 g/dL, 総蛋白 5.3 g/dL，アルブミン 2.2 g/dLであり，白血球数およびリンパ球数低値，貧血および低アルブミン血症を認めた. C反応性蛋白（CRP） 0.3 mg/dL, トランスサイレチン（TTR） 20.1 mg/dL,

マグネシウム 1.9 mg/dL，リン 3.4 mg/dL，銅 80 μg/dL，亜鉛 68 μg/dL，乳酸 11 mg/dLであった.

入院時画像所見：

胸部レントゲン検査（Figure 1a）：両側胸腔内の肺野の均一な透過性の低下を認め，両側に胸水と前医で留置された胸腔両側にドレナージチューブを認めた.

胸部CT検査（Figure 1bc）：中等量の胸水貯留を両側に認めた（前日抜水後）.

入院後経過：主観的包括的アセスメント（subjective global assessment: SGA）では高度栄養不良と評価され，必要予測エネルギー量を1573 kcal（基礎代謝量1093 kcal×活動係数1.2×ストレス係数1.2）と算出した．これに基づき，１日当たり，エネルギー1600 kcal，蛋白65 g（内，分岐鎖アミノ酸, branched-chain amino acid：BCAA 2g），脂質45 g，炭水化物230 g（PFC比＝16：25：58）の摂取を基本計画とした. 胃切除術後で食欲不振もあったため, 食事は軟菜半分食（800 kcal, 蛋白35g）に設定した．本人の嗜好に合わせた食事内容に調整したことにより，入院当初より全量摂取が可能であった. 末梢挿入型中心静脈カテーテル（peripherally inserted central catheter: PICC）を留置し，不足分を中心静脈栄養（高カロリー輸液製剤+脂肪乳剤:760 kcal，アミノ酸20g）で補い，加えて経口栄養補助剤（oral nutritional supplements , ONS） 1/2包（150 kcal，蛋白4.4 g）を付加し，アミノ酸・蛋白質を強化した．1日投与量は，エネルギー1710 kcal（経口栄養950 kcal+静脈栄養760 kcal）, 蛋白59.4 g（内BCAA5.3g, 8.9%）, 脂質40 g, 炭水化物273 g（PFC比＝14:21:65）となった．呼吸困難を伴う両側胸水で既に胸腔チューブが留置されていて，安全に抜水ができることから両側胸水に対してCPRTを施行した.

初回CPRTでは，両側胸水800 mLを回収後120 mLを還流し，2週間後に施行した2回目のCPRTでは，両側胸水460 mLを回収後100 mLを還流した．抜水した胸水は細胞診検査で白血球増多がないこと，細菌がいないこと，血清でないこと，生化学的に問題がないことを確認して還流した.

6週後には胸水が消失したため胸腔チューブを抜去した． 2回目のCPRT後より全身倦怠感などが改善し，入院2週間後よりリハビリテーション療法を開始した（Figure 2）．リハビリテーション治療開始時の評価では，ふらつきがあり歩行はできず，両下肢には浮腫を認めた．徒手筋力テスト（manual muscle test: MMT）の結果はレベル4で，日常生活動作については機能的自立度評価法（functional independence measure: FIM）のセルフケアが4点，移乗能力が3点であり車いす移乗からの開始となった．週1回NST回診を行い，栄養治療計画と評価を行った．この間ダンピング症状のため食事量の増量は難しく，静脈栄養を併用したが体重減少，血液検査での肝機能や中性脂肪の上昇などはみられなかったため，投与栄養量に過不足はないと判断し，入院時に立案した栄養プランを継続した．栄養指標では，入院後2週間で血清TTRが20.7 mg/dLから27.2 mg/dLへと上昇し，それに伴って血清アルブミン値が徐々に上昇し，入院時の2.2 g/dLから2か月後には3.0 g/dLに達した．血清乳酸値も11.2 mg/dLから7.3 mg/dLへと低下した．入院2か月後の画像検査では，胸部単純レントゲン検査およびCT検査で両側ともに胸水貯留を認めなかった（Figure 3）．また，体組成を入院直後と2か月後に体成分分析装置（InBody^{TM}）を用いて測定した．位相角（phase angle）は，浮腫のあった入院時は2.0°であったが，2か月後には2.9°と上昇していた．また浮腫の指標となる細胞外水分量（extracellular water: ECW）/総体水分量（total body water: TBW）比も，0.436と「0.43 以上」の高い判定から，0.423と「0.40 以上～0.43 未満」の「やや高い」へと若干の改善を認めた（Table 1）．その後，胸水の再貯留は認めずリハビリテーション治療にて歩行練習を行い，最終的にMMTの結果は強い抵抗を加えても運動範囲全体にわたって動かすことができるレベル5（正常）となり，日常生活動作についてはFIMのセルフケアが6点，移乗能力が7点，移動能力も杖なしで自立独歩が可能な7点に達し退院となった．

退院後は，実施中の補助的静脈栄養の継続は希望されず，退院後の栄養管理計画として経口摂取のみで療養可能か検討を行った。退院後の必要予測エネルギー量を1429 kcal（基礎代謝量

1099 kcal×活動係数1.3×ストレス係数1.0）と算出した．エネルギー1400 kcal，蛋白60 g，脂質40 g，炭水化物200 g（PFC比＝17:26:57）を摂取できるように計画を立案し退院時の栄養指導を行った．入院中，3食と10時，15時のONSでの補食を本人家族に提案した．少量頻回食の受け入れがあったことと，退院後はすぐ近くの前医に必要時の対応を前提に外来通院することとなり，入院第64日目に独歩退院した.

【考察】

低アルブミン血症に対する治療としては，アミノ酸の投与が蛋白質合成に有効であることから，経腸または静脈栄養法の早期実施が有効であるとしている[4] .自験例においても，早期より経口栄養食の工夫に加えてBCAAを含むONSの補充により改善を認めた.

CARTは1973年に日本で山崎らによって開発され[5]，現在では肝硬変やがん性腹水などで実施されている[2]．CARTは終末期がん患者に対する治療が多く，低栄養患者の難治性腹水に対してCARTを実施している医療機関は少ない[6]．低栄養による胸水であれば早期の適切な栄養管理により改善する可能性が高く，CPRTは即適応となることは少ない．しかし，本例のように高齢者であり常時ドレナージが必要な病状に陥った場合は，早期にドレナージ不要に導き日常生活が維持できるまで回復させることが必要であり，より積極的な胸水治療としてのCPRTは有意義であったと考える．PubMedで発行年を問わずcell-free and concentrated pleural effusion reinfusion therapyで検索したところ2件であり，この内，低栄養に伴うものは１件であった．医中誌でも発行年を問わず栄養障害，難治性胸水で検索した結果論文はなく，胸水濾過濃縮再静注法，胸腹水濾過濃縮再静注法，胸・腹水濾過濃縮再静注法で検索したところ胸水に関するものは３件であった．これらの報告に加えて参考文献より調べた結果，CPRTの報告は全部で6件であり一覧表をtable 2. に示す[7-12]．原因として低栄養が関与した可能性がある報告は3例のみであった.

日本緩和医療学会からは，抜水の量は1000ml程度にすることが推奨されており[13]，これに比べ

てCPRTは血管内浸透圧を上げることができるため一気に大量の抜水も可能で，胸水が残ることがなく呼吸困難や全身倦怠感などの症状が緩和されるため，ADLが上がっていると考えられる．24時間以内に1500mlを超える排液をしないように勧める医師もいるが，排液の量と再膨張性肺水腫のリスクが直接相関することを示すエビデンスはほとんどないことが同じ引用の報告でされており，当科では抜水はできるかぎり行っている[13)]．

肺がんの胸腔内播種に伴う症例[10)]と胸管破綻に伴う乳び胸の症例[9,12)]以外は，低栄養が関与している可能性がある症例報告では、効果があるまで2回までのCPRTであり[7,8,11)]，栄養不良による胸水に対しては自験例のように2回程度のCPRTで改善が見込まれることが示唆された．また，本症例では2週間で血清TTRが20.7 mg/dLから27.2 mg/dLへと上昇しており，栄養状態の早期改善がCPRTが2回で終了できた要因と考えられた．合併症については，これまでの報告で頻回に穿刺を行った症例では気胸を合併していたが，今回のように２回までの胸水穿刺で合併症の報告はなく，安全に施行できると考えられる．腹水の灌流時に予防策としてステロイドを投与している施設もあるが，当科の場合は，終末期のがん患者や低栄養で免疫力の低い患者が多いこともあり，血糖値の変動と免疫の面からもステロイドの予防投与は行ってない．当科でも，頻回の穿刺で軽度気胸を起こした症例が１例のみあるが保存加療で問題なかった．それ以外に気胸はなく，これまで微熱が出る程度で解熱剤の対応で問題なく行っている．今回の栄養不良に伴う難治性胸水を対象とするCPRTは，強化型栄養療法を併用した介入であり，CPRTによる減圧ドレナージによる呼吸器症状の改善および栄養改善効果が相加的に現れたと考えられる．

最近は，代謝異常や栄養不良が進行していない前悪液質のような段階においては，筋肉量の維持あるいは増強を図る負荷運動も推奨されるようになってきている[14,15)]．本症例は，リハビリテーションを行うことで自力歩行が可能となり退院できた．今回リハビリテーションによりMMTは4から5に，FIMはセルフケアが4点から6点，移乗能力が3点から7点となり，移動能力も

立位が出来なかった状態から自立独歩可能な7点となっており，筋肉量としてSMIは7.09から6.85と若干の低下であったが筋力としては改善を認めた．BIAで測定される位相角は，細胞膜の状態を表し栄養失調のバイオマーカーとして用いられており[16)], 電流が体水分に沿って流れる際に発生する抵抗(レジスタンス)と，細胞膜を通過する際に発生する抵抗(リアクタンス)の位相差です．つまり，位相角は細胞膜の健康度・細胞の構造的な安定度合いを反映し，位相角０°は細胞の破壊を意味するので位相角が低いほど細胞の健康状態・機能が低下していることを意味する。本症例では位相角が2.0°から2.9°と改善したが，有効か否かの判断まではできない．ECW/TBWも高い判定からやや高いへと若干の改善を認めた．本例のように入院時に電気インピーダンスで細胞外水分量が多い人，位相角が低い人が時系列的に改善してくるかどうかが奏功するかどうかを判定するのに有効であると考えられる．今回の栄養強化とCPRTおよびリハビリテーション治療は独歩退院のために有効な手法であったと考えられた。

【まとめ】

低栄養を伴う難治性胸水に対し，CPRTと強化型栄養療法は胸水の消失とADLおよび栄養状態の改善に有効である可能性が示唆された．

著者にCOIはありません．

参考文献

1) Ito T, Hanafusa N. CART: Cell-free and Concentrated Ascites Reinfusion Therapy against malignancy-related ascites. Transfus Apher Sci 2017;56:703-7.
2) Chen H, Ishihara M, Horita N, Tanzawa S, Kazahari H, Ochiai R, Sakamoto T, Honda T, Ichikawa Y, Watanabe K, Seki N. Effectiveness of Cell-Free and Concentrated Ascites Reinfusion Therapy in the Treatment of Malignancy-Related Ascites: A Systematic Review and Meta-Analysis. Cancers (Basel) 2021;13:4873.
3) Crawford GB, Robinson JA, Hunt RW, Piller NB, Esterman A. Estimating survival in patients with cancer receiving palliative care: is analysis of body composition using bioimpedance helpful? J Palliat Med 2009;12:1009-14.
4) Muscaritoli M, Arends J, Bachmann P,et al. ESPEN practical guideline: Clinical Nutrition in cancer. Clin Nutr 2021;40: 2898-913.
5) Yamazaki Z. fukusui no roka(jyokinjyogansaibou)nousyukufukusuisaikanryuryouhou. Surgery 1975;37:1628-9 (in japanese).
6) Hanafusa N, Isoai A, Ishihara T, et al. Safety and efficacy of cell-free and concentrated ascites reinfusion therapy (CART) in refractory ascites: Post-marketing surveillance results. PLoS One 2017;12:e0177303.
7) Furukawa H, Miyajima Y, Ueki T, Nakaoka K, Matsuoka M, Sakaguchi M, Okumura M. rokanosyukusaijyocyuho de koka wo mita nanchiseikanseikyosui no ichirei. rinsyotokenkyu 1990;67: 3498-500 (in Japanese).
8) Kurioka Y, Takahashi K, Ozaki T, Ueda T, Miyazaki K. kyo･fukusuirokanosyukusaijyocyuho ga soukou sita jyusyoransokajyosigekisyokogun no ichirei. nihonsanfujinkagakkaicyugokushikokugodochihobukaizasshi 2003;51:178-86(in Japanese).
9) Sawada Y, Nomura Y, Yoshii Y. jyutsugonyubikyo no hojyoryoho toshite kyosuirokanosyukusaijyocyuho ga yuko de atta ichirei (Cell-Free and Concentrated Pleural

Effusion Reinfusion Therapy for Postoperative Chylothorax). Jpn. J. Cardiovasc. Surg 2009;38:205-207(in Japanese).

10) Takeuchi A, Takahashi N, Hiyama N, Watanabe H. kyokunaikateteruhikapotosisutemuryuchika ni okonatta kyosuirokanosyukusaijyocyuho niyori cyokijitakuryoyo ga kano de atta haigan no ichirei(Cell－Free and Concentrated Pleural Effusion Reinfusion Therapy Using an lntrathoracic Catheter Subcutaneous Port System Can Achieve a Long－Term Home Care for a Patient with Refractory Pleural Effusion in Lung Cancer). yamaguchiigaku 2016;65:167-72(in Japanese).

11) Xiaolin Y, Takeda S, Watanabe Y, Iida M, Yamamoto T, Nakashima C, Nishiyama M, Matsui H, Shindo Y, Tokumitsu Y, Tomochika S, Yoshida S, Suzuki N, Ioka T, Nagano H. jyutsugokyosuikontororu ni kuryoshita kankohenheizonkyobusyokudogan no ichisetsujyorei(Postoperative Management of Refractory Pleural Effusion in a Patient with Esophageal Cancer Accompanied by Cirrhosis). Gan To Kagaku Ryoho 2021;48:2036-8(in Japanese with English abstract).

12) Kuwahara Y, Tashiro H, Takeshita G, Egashira Y, Maruyama A, Ikeda Y, Kimura S, Sueoka-Aragane N, Takahashi K. Refractory bilateral chylothorax and chylous ascites in a patient with systemic lupus erythematosus treated by pleuro-peritoneal and peritoneal-venous shunts along with cell-free and concentrated ascites re-infusion therapy. Respiratory Investigation. 62(6):1191-1194. 2024.

13) Feller-Kopman D, Berkowitz D, Boiselle P, et al: Large-volume thoracentesis and the risk of reexpansion pulmonary edema.Ann Thoracic Surg 84:1656–1662, 2007.

14) Skipworth RJ, Stene GB, Dahele M, Hendry PO, Small AC, Blum D, Kaasa S, Trottenberg P, Radbruch L, Strasser F, Preston T, Fearon KC, Helbostad JL. Patient-focused endpoints in advanced cancer: criterion-based validation of accelerometer-based activity monitoring. Clin Nutr 2011;30:812-21.

15) Strasser B, Steindorf K, Wiskemann J, Ulrich CM. Impact of resistance training in cancer

survivors : a meta-analysis. Med Sci Sports Exerc 2013;45:2080-90.

16) Gupta D, Lammersfeld CA, Vashi PG, King J, Dahlk SL, Grutsch JF, Lis CG. Bioelectrical impedance phase angle as a prognostic indicator in breast cancer. BMC Cancer 2008;8:249.

17) Masuda Y, Yokose S, Miyamoto K, Nagata M, Miyamoto J. manseijitosekikanjya no eiyohyoka ni taisuru Phase Angle no ichikosatsu. Journal of Japan Association for Clinical Engineers 2019;65: 88-92 (in Japanese).

Legend for figures

Figure 1　Imaging study on admissin　a；Chest X-ray (supine position), b,c；Chest CT scan.

a: pleural effusion and thoracic drainage tubes were placed on both thoracic cavity (black allow).

b,c: moderately amounts of pleural effusion were observed on both sides.

Figure 2　Progress after admission

Figure 3　Imaging study 2 months after admission: a: Plain chest X-ray (standing position), b: Chest CT scan. a,b: No pleural effusion was observed on either side.

Table 1　Changes in nutritional assessment have obtained from the body composition analyzer (InBody^{TM}).　Phase angle and ECW/TBW have been improved
